# Effectiveness of highand medium-fidelity simulation in nursing education: randomized controlled trial

**DOI:** 10.1590/0034-7167-2025-0134

**Published:** 2026-03-30

**Authors:** Franciane Silva Luiz, Isabel Cristina Gonçalves Leite, Luana Vieira Toledo, Kelli Borges dos Santos, André Luiz Silva Alvim, Roberta Teixeira Prado, Angélica da Conceição Oliveira Coelho, Fábio da Costa Carbogim

**Affiliations:** I Universidade Federal de Juiz de Fora. Juiz de Fora, Minas Gerais, Brazil; II Universidade Federal de Viçosa. Viçosa, Minas Gerais, Brazil

**Keywords:** Simulation Training, High Fidelity Simulation Training, Education, Nursing, Clinical Competence, Pressure Ulcer., Entrenamiento Simulado, Enseñanza Mediante Simulación de Alta Fidelidad, Educación en Enfermería, Competencia Clínica, Úlcera por Presión.

## Abstract

**Objectives:**

to compare the effectiveness of highand medium-fidelity clinical simulation on nursing students’ knowledge and clinical skills for pressure injury assessment and treatment.

**Methods:**

assessor-blinded randomized controlled trial. Thirty-two nursing students were assigned to an experimental group (n=17) or a control group (n=15). The intervention comprised a theoretical component and a simulation-high fidelity for the experimental group and medium fidelity for the control group. Data were collected with a sociodemographic questionnaire, a theoretical test, a skills checklist, and a Debriefing Assessment Scale. Statistical analysis included the chi-square test, the Mann-Whitney U test, ANOVA, and effect size, at a 5% significance level (α=0.05).

**Results:**

both groups showed significant improvement in theoretical knowledge (p<0.05). The groups did not differ significantly in skills (p=0.853). The debriefing experience was positive and similar in both groups.

**Conclusions:**

simulation improved knowledge and skills, with no differences by simulation fidelity level.

## RESUMEN

**Objetivos:**

comparar la eficacia de la simulación clínica de alta y media fidelidad en el desarrollo de conocimientos y habilidades de estudiantes de enfermería para la valoración y tratamiento de lesiones por presión.

**Métodos:**

ensayo clínico aleatorizado con cegamiento del evaluador. Se asignó aleatoriamente a 32 estudiantes a grupo experimental (n=17) o control (n=15). Intervención: componente teórico más simulación de alta fidelidad (experimental) o media fidelidad (control). Recogida de datos mediante cuestionario sociodemográfico, prueba teórica, lista de verificación de habilidades y Escala de Evaluación del Debriefing Asociado a la Simulación. Análisis: chi-cuadrado, U de Mann-Whitney, ANOVA y tamaño del efecto; α=0,05.

**Resultados:**

hubo mejora significativa del conocimiento teórico en ambos grupos (p<0,05). No hubo diferencias entre grupos en habilidades (p=0,853). La experiencia de debriefing fue positiva y similar.

**Conclusiones:**

la simulación mejoró conocimiento y habilidades, sin diferencias por nivel de fidelidad.

## INTRODUCTION

Clinical simulation has emerged as an effective and widely used educational strategy in nursing education because it enables active, safe, student-centered learning^(1)^. By allowing students to engage with complex clinical situations in a controlled environment, simulation fosters the development of both technical and nontechnical skills-decision-making, clinical reasoning, communication, and teamwork^(2)^. Its use in teaching has substantially contributed to consolidating knowledge and enhancing the skills required for high-quality professional practice^(2,3)^.

Traditionally, simulation scenarios have been classified by fidelity level (low, medium, or high), considering realism and the technology employed^(2)^. However, contemporary approaches emphasize that effectiveness depends more on participant immersion, alignment with learning objectives, and the quality of instructional design than on the technological level or manikin complexity alone^(2-4)^. Accordingly, high-quality simulations can be delivered with simple resources when they are carefully planned and aligned with educational goals^(3-5)^.

Within nursing education, simulation has been used to promote clinical skill development across several areas, including the prevention and management of pressure injuries (PI)^(6)^. PI-localized damage to the skin and/or underlying tissues from prolonged pressure or shear-is a key indicator of the quality of care and is highly prevalent among hospitalized patients^(5,6)^. Despite well established prevention guidelines, such as repositioning, the use of support surfaces to redistribute pressure, and the application of risk assessment scales, translating theoretical knowledge into clinical practice remains a significant challenge^(5)^.

It is therefore essential for nursing students to develop clinical skills for effective PI assessment and management. Evidence-based active teaching strategies, such as clinical simulation, are widely recommended because they provide safe, controlled environments for practicing decision-making and problem-solving, as well as implementing care interventions^(7,8)^.

Previous studies have reported mixed findings on clinical simulation for PI education^(9-11)^. One study^(12)^ involving 47 Chinese nursing students found that simulation was more effective than traditional teaching for addressing PI. Conversely, an experimental study with 84 Turkish students found no significant differences in knowledge before and after a simulation intervention^(13)^. However, given the scarcity of investigations that compare different simulation fidelity levels in terms of nursing knowledge and skill acquisition, additional studies are warranted.

Against this backdrop, the following question arises: In nursing education, is a high-fidelity clinical scenario more effective than a medium-fidelity one for developing knowledge and skills for PI assessment and treatment?

## OBJECTIVES

To compare the effectiveness of highand medium-fidelity clinical simulation in developing nursing students’ knowledge and clinical skills for the assessment and treatment of pressure injuries.

Consistent with the study objective, we hypothesized that the high-fidelity clinical scenario would lead to greater improvement in clinical skills for PI management.

## METHODS

### Ethical aspects

The study adhered to Brazilian National Health Council (CNS) Resolution 466/12 and used an Informed Consent Form (ICF). It was approved by the Research Ethics Committee of the Federal University of Juiz de Fora.

### Study design, period, and setting

Assessor-blinded randomized controlled trial with parallel groups, conducted in accordance with the Consolidated Standards of Reporting Trials (CONSORT)^(14)^. The CONSORT checklist is available as supplementary material.

The study was carried out in August 2023 at a public college in the state of Minas Gerais, Brazil, and the protocol was registered in the Brazilian Clinical Trials Registry (ReBEC) under the code RBR-7w6dh9q.

### Population and sample

A nonprobability, purposive sample was used, comprising 32 students enrolled in the sixth semester of the undergraduate nursing program who consented to participate. This semester was selected based on the program’s pedagogical plan, as students had already completed the course in health assessment and nursing procedures, which is essential to understand the content addressed in the PI intervention. At the institution studied, the nursing program lasts five years, organized into ten semesters, with up to 40 students admitted each semester.

Exclusion criteria were prior completion of the specific content addressed, suspended enrollment or leave of absence, and failure to complete the intervention in full.

Although the sample size (n = 32) is small, it comprised all students officially enrolled and present in the target class at the time of data collection and therefore reflected the entire cohort available for the proposed intervention. No a priori sample size calculation was performed because the study was exploratory and conducted in a specific context involving the accessible population of eligible students. Including all class members ensured complete coverage of the target population and allowed assessment of feasibility and the initial effects of the proposed educational strategy.

### Randomization and blinding

Stratified randomization assigned the 32 students to the experimental and control groups (1:1) to ensure balance in baseline characteristics. Strata were defined by sex (male/female), age group (five-year bands), and study shift (morning/afternoon). An independent statistician-uninvolved in recruitment, intervention delivery, or data collection-generated the randomization sequence. For each stratum, the statistician used the Microsoft Excel 2010 “RAND ( )” function to generate a random value for each participant, ordered participants by that value, and assigned them 1:1 in sequence (Group A = experimental, Group B = control).

The final sequence was coded (Group A/Group B) and stored in a password-protected electronic file accessible only to the statistician. Allocation concealment was ensured through a centralized procedure: investigators who enrolled participants requested assignment from the statistician only after confirming eligibility and obtaining the signed ICF; the statistician then returned only the allocation code (A or B). Thus, the recruitment team and the professionals who delivered the interventions had no prior access to the allocation list.

Regarding blinding, outcome assessors and the team responsible for developing the instructional materials did not take part in intervention delivery or data collection; assessor blinding was therefore maintained. Participants were not informed of the comparative nature of the groups (partial participant blinding), and the professionals who delivered the interventions worked without access to the group composition. In summary, the trial used stratified randomization with the sequence generated by an independent statistician and centralized allocation concealment. Blinding was maintained for assessors/analysts, with partial participant blinding, but not for intervention providers.

In accordance with CONSORT, we explicitly report: 1) sequence generation-Excel “RAND ( )” function by an independent statistician, 2) allocation concealment-a coded, password-protected file with assignment communicated only after inclusion, 3) implementation-the statistician generated and held the list, investigators enrolled participants, the statistician communicated assignments, and a team delivered the interventions without access to the list.

### Data collection instruments and variables

Data were collected using a sociodemographic questionnaire, a theoretical knowledge test^(15)^, a simulated clinical scenario with a skills checklist^(16)^, and the Simulation Debriefing Assessment Scale (SDAS)^(17)^.

The sociodemographic questionnaire included age, sex, race/ethnicity, semester in the program, prior training in health, and prior exposure to the topic. The theoretical knowledge test comprised 20 items on the prevention and management of PI, with response options True (T), False (F), or Not sure (NS); each correct answer awarded 1 point. In validation, the test showed Cronbach’s alpha of 0.85.

The simulation employed a structured, content-validated clinical scenario that addressed PI treatment in a hospital clinical care setting. The structured scenario demonstrated an overall Content Validity Index (CVI) of 0.85. The skills checklist^(16)^ used in the scenario contains 13 items that assess the student’s ability to identify, assess, select, and indicate the appropriate wound dressing. Each item was scored as Not performed (0 points), Partially performed (0.5 point), or Performed adequately (1 point). To achieve the minimum required score of 70%, students needed ≥ 9.1/13 points^(15)^. Notably, the checklist is one component of the structured clinical scenario.

To assess the impact of debriefing from the participants’ perspective, we used the SDAS^(17)^, a 32-item instrument organized into three dimensions: Psychosocial value dimension, Cognitive value dimension, and Affective value dimension. The scale showed high internal consistency (Cronbach’s alpha = 0.899).

The primary outcome was adequate PI management, measured with the scenario skills checklist. Secondary outcomes were theoretical knowledge and the impact of debriefing, assessed with the theoretical knowledge test and the SDAS, respectively.

### Study protocol: intervention and data collection

The study comprised six stages: 1) collection of sociodemographic data and assessment of knowledge before the educational intervention (pretest); 2) theoretical instruction on PI; 3) skills training with clinical photographs of PI for both the control group (CG) and experimental group (EG); 4) intervention: implementation of the clinical scenario and assessment of skills using the checklist; 5) debriefing and administration of the Simulation Debriefing Assessment Scale (SDAS); and 6) assessment of knowledge (posttest).

In stage one, students completed a sociodemographic questionnaire and took the initial theoretical knowledge test on PI (pretest) in a private room for 30 minutes. In stage two, the theoretical content was delivered over eight hours by ostomy-care nurses experienced in clinical practice and teaching. The instruction covered three major topics: Introduction to wounds, Introduction to PI, and Assessment and treatment of these injuries. This stage took place on the same day as stage one. On the following day, stage three was conducted, during which participants underwent hands on training with photographs of PIs. The training, identical for both groups, lasted two hours and aimed to familiarize students with PI classification and assessment.

Stage four, conducted 14 days after stage one, was the intervention phase, during which the clinical simulation was carried out and skills were assessed. The two-week interval was adopted to minimize recall bias from the previous test, ensuring that performance on the second test reflected actual knowledge retention. At this stage, four nurses (two for EG and two for CG) provided scenario guidance and conducted the assessment using a validated checklist. The stage began with a 5-minute briefing, followed by 12 minutes for each student to complete the activities in the simulated scenario.

The intervention for EG consisted of a high-fidelity scenario characterized by greater realism and interactivity. To this end, clinical simulation employed a standardized patient portrayed by a trained actor who interacted with students; moulage (artificial wounds) was applied to the skin, and clinical cases were presented. The CG underwent a medium-fidelity scenario that used a Pressure Injury Stage Simulator manikin (TZJ-4009-U), also in conjunction with clinical cases. Accordingly, by including a trained actor that enabled realistic interaction, the high-fidelity scenario provided a more immersive experience than the medium-fidelity environment, which featured a lower level of interactivity.

Both the nurses and the actor underwent specific, standardized training based on a detailed protocol. This training ensured a clear understanding of objectives, required actions, and procedures. Nurses assigned to the EG were trained separately from those assigned to the CG to avoid cross influence. The instructional style was standardized-encompassing the language used, time allotted to each stage, and feedback approach-to minimize individual differences among instructors and ensure comparable delivery across groups. This careful, coordinated calibration ensured a controlled intervention with maximum fidelity to the protocol, providing participants with an immersive, high-quality learning experience.

The fifth stage (debriefing) was conducted at the end of the educational intervention, separately for EG and CG in distinct rooms. Participants were taken to the debriefing room and remained there until the group was complete. This 30-minute stage was led by the same nurses who had assessed the skills in the clinical scenario and followed a structured script with guiding prompts^(18)^. Ultimately, students completed the Simulation Debriefing Assessment Scale (SDAS)^(17)^, which took an average of 20 minutes.

The sixth and final stage consisted of administering the posttest to assess theoretical knowledge about PI. Students completed the same test used in stage one, in a private room, for 30 minutes.

Across the six stages, each participant completed approximately 12 hours of activities. All elements of the intervention were standardized in advance through specific training for the nurses and the actor to ensure protocol fidelity and consistency of assessments.

### Data analysis

Data were tabulated in Microsoft Excel^®^ and analyzed in R 4.3.0. Categorical variables were summarized as absolute and relative frequencies, whereas numeric variables were summarized as mean (with 95% CI), median, standard deviation, quartiles, and minimum-maximum. Internal consistency was assessed with Cronbach’s alpha and McDonald’s omega, and inter-item correlations were computed using the Pearson correlation coefficient.

Between-group comparisons (EG vs CG) used the chi-square test (or Fisher’s exact test) for categorical variables and Student’s t test or the Mann-Whitney U test for numeric variables, as appropriate to distributional assumptions. Changes over time were examined with mixed-model ANOVA.

Effect sizes (Cramér’s V, Cohen’s d, r, and η^2^) were calculated to assess the magnitude of results, using a 5% significance level (α=0.05).

## RESULTS

**Figure 1:**
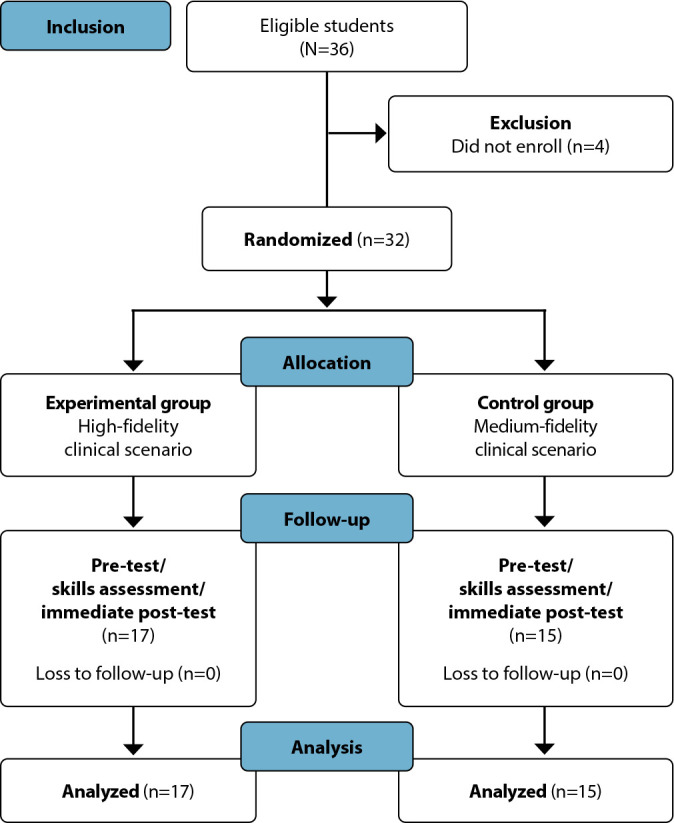
Randomized controlled trial flow diagram according to the Consolidated Standards of Reporting Trials (CONSORT)^(14)^ (n = 32), Minas Gerais, Brazil, 2023

Of the 36 eligible students, 32 completed the intervention protocol. Randomization assigned 17 students to the EG and 15 to the CG, with no loss to follow-up (Figure 1).

Most eligible students were female (71.9%), with a mean age of 22.56 years (SD = 1.80; 95% CI 21.87-23.25). The groups were homogeneous, with no differences in sociodemographic characteristics or prior exposure to the topic (Table 1).

**Table 1:** Comparison of sociodemographic variables and prior exposure between the experimental and control groups (N = 32), Minas Gerais, Brazil, 2023

Variable	Group		*p* value
	Control group (n = 15)	Experimental group (n = 17)	
Age (SD^*^)	22.67 (1.80) IC 95%^†^: 21.56-23.77	22.47 (1.91) IC 95%^†^: 21.25-23.68	0.768‡
**Sex - n (%)**			
Female	11 (73.33)	12 (70.59)	1.000^§^
Male	4 (26.67)	5 (29.41)	
**Race - n (%)**			
White	10 (66.67)	10 (58.82)	
Mixed (Pardo)	3 (20.00)	5 (29.41)	0.875^§^
Black	2 (13.33)	2 (11.76)	
**Has technical-level training in health - n (%)**			
No	14 (93.33)	17 (100.00)	0.469^§^
Yes	1 (6.67)	0 (0.00)	
**Prior exposure to the topic - n (%)**			
No	15 (100.00)	17 (100.00)	0.456^§^
Yes	0 (0.00)	0 (0.00)	

* Standard deviation;

† 95% CI;

‡ Student’s t test for independent samples;

§ Fisher’s exact test.

On the 20-item theoretical knowledge test, the pretest median was 12 for the CG and 13 for the EG; in the posttest, both groups had a median of 15 (Table 2).

**Table 2:** Descriptive statistics for correct answers on the knowledge questionnaire, by group and time point in the pretest and posttest (N = 32), Minas Gerais, Brazil, 2023

Variable	Pretest		Posttest	
	Control group (n = 15)	Experimental group (n = 17)	Control group (n = 15)	Experimental group (n = 17)
Number of correct answers				
Mean (SD^*^)	12.47 (2.61)	12.12 (1.96)	15.36 (1.36)	15.07 (2.37)
95% CI^†^	11.06-13.87	10.99-13.26	14.53-16.18	13.84-16.31
Median (Q1 - Q3)‡	12.00 (10.50 - 15.00)	13.00 (10.00 - 14.00)	15.00 (14.00 - 16.00)	15.00 (13.25 - 16.75)
Min. - Max.^§^	8 - 16	8 - 15	14 - 18	11 - 19

* Standard deviation;

† 95% CI;

‡ Q1 = first quartile (25th percentile); Q3 = third quartile (75th percentile);

§ Observed minimum and maximum values.

Mixed-model ANOVA showed a significant increase in correct answers from pretest to posttest, irrespective of group (Time factor: F(1,23) = 25.298; p < 0.001; η^2^g = 0.333), indicating a substantial change in scores over time.

In addition, no significant Group × Time interaction was identified (F(1,23) = 0.154; p = 0.698; η^2^g = 0.003), indicating no between-group difference in overall performance. In addition, no significant Group × Time interaction was identified (F(1,23) = 0.154; p = 0.698; η^2^g = 0.003), indicating that the increase in correct answers from the pretest to the posttest occurred similarly in both groups, with no variation attributable to the interaction between these factors.

Accordingly, the improvement observed in the number of correct answers is attributable to the temporal factor (assessment time), with no significant influence of group or the Group × Time interaction. These findings indicate similar performance trajectories in both groups and suggest that factors other than group assignment may have contributed to the increase in correct answers. Moreover, internal consistency of the PI knowledge test was adequate (Cronbach’s alpha = 0.705; McDonald’s ω = 0.798), reinforcing the reliability of the assessment.

For the skills assessment in the simulated clinical scenario, overall scores were very similar between groups, with means of 10.91 (CG) and 10.79 (EG). The EG performed better on Item 2 (dressing selection) and Item 11 (wound edges), but showed greater difficulty on Item 5 (anatomic location). The CG exhibited a more homogeneous performance, except for Item 9 (pain classification) (Table 3).

**Table 3:** Descriptive analysis of skill-related variables in the simulated clinical scenario, by group (N = 32), Minas Gerais, Brazil, 2023

Variable	Group (N = 32)	
	Control group (n = 15) - n (%)	Experimental group (n = 17) - n (%)
Item 1: Did students identify and stage the pressure injury?		
Correct	12 (80.00)	15 (88.30)
Incorrect	3 (20.00)	2 (11.70)
**Item 2: Did students select and report appropriate wound dressings?**		
Correct	13 (86.70)	17 (100.00)
Incorrect	2 (13.30)	0 (0.00)
**Item 3: Did students justify the choice (indication) of wound dressings?**		
Correct	13 (86.70)	14 (83.35)
Incorrect	2 (13.30)	3 (17.65)
**Item 4: Did students instruct the patient/family about the dressing and other procedures that could improve the overall PI condition?**		
Correct	15 (100.00)	17 (100.00)
Incorrect	0 (0.00)	0 (0.00)
**Item 5: Did students identify and classify the anatomic location of the pressure injury?**		
Incorrect	3 (20.00)	12 (70.60)
Correct	12 (80.00)	5 (29.40)
**Item 6: Did students document the wound size (length, width, and depth)?**		
Correct	15 (100.00)	17 (100.00)
Incorrect	0 (0.00)	0 (0.00)
**Item 7: Did students assess and classify the tissues present in the pressure-injury bed?**		
Correct	15 (100.00)	16 (94.10)
Incorrect	0 (0.00)	1 (5.90)
**Item 8: Did students identify and classify the types of wound dressings?**		
Correct	15 (100.00)	16 (94.10)
Incorrect	0 (0.00)	1 (5.90)
**Item 9: Did students identify and classify pain?**		
Incorrect	13 (86.70)	12 (70.60)
Correct	2 (13.30)	5 (29.40)
**Item 10: Did students identify and classify the condition of the periwound skin?**		
Correct	12 (80.00)	14 (83.35)
Incorrect	3 (20.00)	3 (17.65)
**Item 11: Did students identify and classify the wound edges?**		
Correct	10 (66.70)	15 (88.30)
Incorrect	5 (33.30)	2 (11.70)
**Item 12: Did students identify and classify exudate characteristics?**		
Correct	13 (86.70)	12 (70.60)
Incorrect	2 (13.30)	5 (29.40)
**Item 13: Did students complete and record the nursing note?**		
Correct	11 (100.00)	12 (70.60)
Incorrect	0 (0.00)	5 (29.40)

Inferential analysis found no statistically significant difference in skills scores between the EG and CG (p = 0.853; small effect size, d = 0.076). Mean scores were similar: 10.91 (SD±1.64; 95% CI 9.96-11.86) in the CG and 10.79 (SD±1.63; 95% CI 9.88-11.71) in the EG. Medians were likewise equivalent: 11.00 (Q1 = 10.00; Q3 = 12.00) in the CG and 10.50 (Q1 = 10.00; Q3 = 12.00) in the EG.

These findings suggest that, despite the interventions, there were no meaningful between group differences in the execution of the assessed skills. Regardless of whether the scenarios were highor medium-fidelity, performance was consistently high, indicating that both groups achieved satisfactory levels in the simulated clinical environment.

For the overall SDAS score, the EG showed a slightly higher mean (121.08±11.22; 95% CI 110.46-121.19) than the CG (115.82±9.03; 95% CI 114.99-127.18). However, the difference was not significant (p = 0.2251), and the effect size was small (d = -0.511). These results indicate similar debriefing experiences across groups, with no clear differences in psychosocial, cognitive, affective, or overall perceptions (Table 4).

**Table 4:** Comparison of SDAS factor scores and overall score between the control and experimental groups (n = 32), Minas Gerais, Brazil, 2023

Dimensions	Group		*p* value	ES^†^
	Control group (n = 15)	Experimental group (n = 17)		
Psychosocial value			0.080^*^	-0.737
Mean (DP^‡^)	54.27 (7.20)	58.64 (4.73)		
95% CI^§^	49.97-58.57	56.14-61.14		
Median (Q1; Q3^||^)	53.00 (51.00; 58.50)	60.00 (57.50; 62.00)		
**Cognitive value**			0.978^#^	-0.011
Mean (DP^‡^)	42.27 (3.29)	42.57 (2.41)		
95% CI^§^	40.45-44.09	41.25-43.89		
Median (Q1; Q3^||^)	44.00 (40.00; 44.50)	43.50 (41.00; 44.75)		
**Affective value**			0.839^#^	0.048
Mean (DP^‡^)	19.27 (6.34)	19.77 (8.22)		
95% CI^§^	15.79-22.75	15.45-24.09		
Median (Q1; Q3^||^)	18.00 (14.00; 24.00)	19.00 (15.00; 20.00)		
**Overall across the three dimensions**			0.225^*^	-0.511
Mean (DP^‡^)	115.82 (9.03)	121.08 (11.22)		
95% CI^§^	110.46-121.19	114.99-127.18		
Median (Q1; Q3^||^)	115.00 (110.50; 121.00)	119.00 (114.00; 125.00)		

* Student’s t test for independent samples;

† Effect size (ES); Cohen’s d for t tests; r for the Mann-Whitney test;

‡ Standard deviation;

§ 95% CI;

|| Q1=first quartile (25th percentile); Q3=third quartile (75th percentile);

# Mann Whitney test.

## DISCUSSION

In this study, the majority of participants were young, female, and White, with some prior exposure to the topic. These characteristics align with findings from other studies that used simulation to teach PI management to undergraduate nursing students^(19-21)^. In another experimental study^(22)^ that also employed clinical simulation for this purpose, the mean age was 21.44 (±2.37) years; 45.7% of the participants were female, and 76.5% reported prior knowledge of the topic.

There was a significant improvement from pretest to posttest in theoretical knowledge in both groups, which likely reflects the combined effect of the theoretical component and clinical simulation. Even so, a systematic review reported no significant knowledge gains with clinical simulation^(21)^. By contrast, a study comparing highand low-fidelity simulations found significant pre-to-post increases in theoretical scores in both groups, corroborating our findings^(22)^.

Theoretical knowledge is pivotal to the effectiveness of clinical simulation because it underpins the psychomotor skills being assessed^(20,21,23)^. It supports rapid, accurate decision-making and strengthens problem-solving, thereby contributing to safer, more effective clinical performance^(21)^. A solid theoretical base also enables students to critically interpret simulated scenarios, apply protocols with confidence, and transfer learning to real clinical settings, thereby reinforcing their professional confidence and competence^(13,21-23)^. Integrating theory and practice is, therefore, essential to maximize the benefits of simulation-based educational strategies.

Regarding clinical skills for PI management, no statistically significant differences were observed between the EG and CG. Despite the greater realism of the high-fidelity scenario (EG), the fidelity level did not affect performance. A plausible explanation is a ceiling effect, given the high and relatively homogeneous performance across groups, which may have limited the ability to detect differences. Thus, the hypothesis that high-fidelity simulation would outperform medium-fidelity simulation in stimulating clinical skills for PI management was not supported.

A study^(24)^ comparing highand medium-fidelity scenarios for PI staging among Turkish nursing students found improvements in theoretical knowledge in both groups; however, the high-fidelity group, which used simulators with wound moulage, achieved significantly higher practical performance and accuracy (p = 0.02). Likewise, a systematic review^(25)^ highlighted that high-fidelity simulation can enhance critical thinking, clinical judgment, and decision-making in nursing students.

However, multiple studies indicate that learning effectiveness in simulation depends more on thoughtful planning and adaptation to learners’ needs than on fidelity level *per se* ^(21,26)^. Clear objectives, alignment with competencies, high-quality briefing and debriefing, and well designed scenarios are more decisive for success than technological realism^(26-30)^.

In debriefing-the final stage of clinical simulation-students reflected on their actions and decisions, promoting self-assessment and integration of theory and practice. We used a structured model that supported reflective review, performance evaluation, and the identification of areas for improvement^(18)^. A systematic review^(29)^ reported that debriefing improves clinical reasoning, critical thinking, self-confidence, and self-efficacy among nursing students.

To assess students’ debriefing experience, we administered the SDAS^(17)^, which examines Psychosocial value, Cognitive value, and Affective value dimensions. Scores were positive across all dimensions, with notable gains in self-confidence, leadership, teamwork, cognitive reflection, and emotion regulation. However, there were no statistically significant differences between the EG and CG, indicating similar benefits across interventions.

### Study limitations

This study has limitations that should be taken into account when interpreting the findings. The small sample and single-center design with a fixed team of faculty/instructors limit generalizability; therefore, extrapolation to other populations and settings should be approached with caution. Nevertheless, the findings offer preliminary evidence of the intervention’s potential and may inform practical application in comparable contexts.

The short follow-up-posttest administered immediately after the intervention with no later skills reassessment-precluded analysis of effect durability. This fact limits the understanding of knowledge consolidation and skill retention over time.

Accordingly, although the results suggest benefits of the strategy used, future studies should increase sample size, include multiple training centers with instructor variability, and adopt a longitudinal design with longer-term follow-up (e.g., 30 or 90 days) to evaluate more robustly the effectiveness and sustainability of educational interventions in this context.

### Contributions to the field

The findings reinforce the relevance of evidence-based educational practices, particularly clinical simulation in nursing education. By comparing different simulation fidelity levels, the results suggest promising directions for refining pedagogical strategies, highlighting the feasibility of lower technology interventions that still support effective learning. This perspective broadens access to active methodologies in resource-constrained settings, contributing to more equitable and sustainable training practices. Despite the limitations, the data provide practical guidance for educational planning and support administrators and educators in adopting realistic, adaptable approaches across diverse nursing education contexts.

For future research, we suggest incorporating qualitative analyses of debriefings to deepen understanding of students’ reflective processes and to identify elements that may further enhance the development of clinical and critical-thinking skills.

## CONCLUSIONS

This study compared the effectiveness of highand medium-fidelity clinical simulation for nursing students in the assessment and treatment of pressure injury. Both groups showed significant gains in theoretical knowledge from pretest to posttest. No statistically significant between-group differences were found in skills performance, with similar scores among students who completed activities in the highand medium-fidelity scenarios. In addition, the debriefing experience was positive and comparable across groups, indicating that critical reflection and immediate feedback were central elements of learning.

## Data Availability

The research data are available within the article.
