# Supplementary material for: Effectiveness of highand medium-fidelity simulation in nursing education: randomized controlled trial
Source: Rev Bras Enferm. 2026 Mar 30;79:e20250134. doi: 10.1590/0034-7167-2025-0134 (PMC13053011; doi:10.1590/0034-7167-2025-0134)
Supplement: Translation

# Translation: Eficácia da simulação de alta e média fidelidade na educação em enfermagem: ensaio clínico randomizado

**DOI:** 10.1590/0034-7167-2025-0134pt

**Authors:** Franciane Silva Luiz, Isabel Cristina Gonçalves Leite, Luana Vieira Toledo, Kelli Borges dos Santos, André Luiz Silva Alvim, Roberta Teixeira Prado, Angélica da Conceição Oliveira Coelho, Fábio da Costa Carbogim

**Affiliations:** I Universidade Federal de Juiz de Fora. Juiz de Fora, Minas Gerais, Brasil; II Universidade Federal de Viçosa. Viçosa, Minas Gerais, Brasil

**Keywords:** Treinamento por Simulação, Treinamento com Simulação de Alta Fidelidade, Educação em Enfermagem, Competência Clínica, Lesão por Pressão.

## Abstract

**Objetivos:**

comparar a eficácia da simulação clínica de alta e média fidelidade no desenvolvimento de conhecimentos e habilidades de estudantes de Enfermagem para avaliação e tratamento de lesões por pressão.

**Métodos:**

ensaio clínico randomizado, com cegamento do avaliador. Alocaram-se 32 estudantes em grupo experimental (n=17) e controle (n=15). A intervenção envolveu abordagem teórica, simulação de alta fidelidade (grupo experimental) e média fidelidade (grupo-controle). Coletaram-se os dados usando questionário sociodemográfico, teste teórico, checklist de habilidades e Escala de Avaliação do *Debriefing*. A análise dos dados incluiu qui-quadrado, Mann-Whitney, Anova e tamanho de efeito, com significância de 5%.

**Resultados:**

houve melhora significante no conhecimento teórico dos dois grupos (p<0,05). Os grupos não diferiram significantemente nas habilidades (p=0,853). A experiência com *debriefing* foi positiva e semelhante em ambos**. Conclusões:** a simulação melhorou o conhecimento e as habilidades, sem diferenças no grau de fidelidade da simulação.

## INTRODUÇÃO

A simulação clínica tem se destacado como uma estratégia educacional eficaz e amplamente utilizada na formação em enfermagem, por possibilitar a aprendizagem ativa, segura e centrada no estudante^(1)^. Ao permitir a vivência de situações clínicas complexas em um ambiente controlado, a simulação favorece o desenvolvimento de competências técnicas e não técnicas, como tomada de decisão, raciocínio clínico, comunicação e trabalho em equipe^(2)^. Sua aplicação no ensino tem contribuído consideravelmente para a consolidação de conhecimentos e aprimoramento das habilidades necessárias à prática profissional qualificada^(2,3)^.

Tradicionalmente, os cenários de simulação têm sido classificados com base no grau de fidelidade (baixa, média ou alta), considerando o realismo e os recursos tecnológicos empregados^(2)^. No entanto, abordagens contemporâneas destacam que a efetividade da simulação está mais relacionada à imersão dos participantes, à congruência com os objetivos de aprendizagem e à qualidade do *design* instrucional do que exclusivamente ao nível tecnológico ou à complexidade do manequim utilizado^(2-4)^. Nesse sentido, simulações de alta qualidade podem ser realizadas com recursos simples, desde que cuidadosamente planejadas e alinhadas às finalidades educativas^(3-5)^.

No contexto da formação em enfermagem, a simulação tem sido utilizada para promover o desenvolvimento de competências clínicas em diferentes áreas, incluindo a prevenção e o manejo de lesões por pressão (LP)^(6)^. Essas lesões, definidas como danos localizados na pele e/ou tecidos subjacentes resultantes da pressão prolongada ou de forças de cisalhamento, constituem importantes indicadores da qualidade do cuidado, sendo altamente prevalentes em pacientes hospitalizados^(5,6)^. Apesar da existência de diretrizes bem estabelecidas para sua prevenção, como a mudança de decúbito, o uso de superfícies especiais para redistribuição da pressão e a aplicação de escalas preditivas de risco, ainda é um desafio significativo transpor o conhecimento teórico para a prática clínica^(5)^.

Diante disso, torna-se fundamental que estudantes de Enfermagem desenvolvam competências clínicas para atuar com eficácia na avaliação e na intervenção diante das LPs. Para tanto, estratégias de ensino ativas e baseadas em evidências, como a simulação clínica, são amplamente recomendadas, pois oferecem ambientes seguros e controlados para a prática da tomada de decisão, da resolução de problemas e da execução de condutas assistenciais^(7,8)^.

Estudos prévios apresentaram resultados divergentes quanto à aplicação da simulação clínica no ensino sobre LP^(9-11)^. Uma pesquisa^(12)^ realizada com 47 estudantes de Enfermagem chineses demonstrou que a simulação foi mais eficaz do que o ensino tradicional na abordagem das LPs. Por outro lado, uma pesquisa experimental conduzida com 84 estudantes turcos não encontrou diferenças significantes nos níveis de conhecimento antes e depois da intervenção com simulação^(13)^. No entanto, como ainda são escassas as investigações que comparam o impacto de diferentes níveis de fidelidade da simulação na aquisição de conhecimentos e habilidades em enfermagem, justificam se estudos adicionais sobre o tema.

Diante desse panorama, indaga-se: No ensino de enfermagem, um cenário clínico de alta fidelidade é mais eficaz que o de média fidelidade no desenvolvimento de conhecimentos e habilidades para avaliar e tratar LPs?

## OBJETIVOS

Comparar a eficácia da simulação clínica de alta e média fidelidade no desenvolvimento de conhecimentos e habilidades de estudantes de Enfermagem para avaliação e tratamento de lesões por pressão.

Assim, consonante ao objetivo do estudo, foi definida como hipótese: o cenário clínico de alta fidelidade propiciará maior estímulo às habilidades clínicas para o manejo de LPs.

## MÉTODOS

### Aspectos éticos

A pesquisa seguiu a Resolução 466/12 do Conselho Nacional de Saúde (CNS), com uso de Termo de Consentimento Livre e Esclarecido (TCLE). Foi aprovada pelo Comitê de Ética em Pesquisa da Universidade Federal de Juiz de Fora.

### Desenho, período e local do estudo

Ensaio clínico randomizado, duplo-cego, com grupos paralelos, conduzido de acordo com as diretrizes do *Consolidated Standards of Reporting Trials* (CONSORT)^(14)^. O checklist está disponível como material suplementar.

O estudo foi realizado em agosto de 2023, em uma faculdade pública do interior de Minas Gerais, Brasil, e o protocolo foi registrado no Registro Brasileiro de Ensaios Clínicos (ReBEC) sob o código RBR-7w6dh9q.

### População e amostra

O estudo utilizou uma amostra não probabilística e intencional, composta por 32 estudantes matriculados no 6º semestre do curso de Enfermagem, que consentiram em participar da pesquisa. A escolha desse semestre foi fundamentada no Projeto Pedagógico do Curso, tendo em vista que os estudantes já haviam concluído a disciplina de Semiologia e Semiotécnica, considerada essencial para a compreensão dos conteúdos abordados na intervenção sobre LP. Na instituição investigada, o curso de graduação em Enfermagem tem duração de cinco anos, organizados em dez semestres, com ingresso semestral de até 40 estudantes.

Foram excluídos os alunos que já haviam cursado previamente o conteúdo abordado, aqueles com matrícula trancada ou afastados, bem como os que não participaram integralmente da intervenção.

Embora o número de participantes (n = 32) possa ser considerado reduzido, ele correspondeu à totalidade de estudantes regularmente matriculados e presentes na turma-alvo no momento da coleta de dados, sendo, portanto, representativo do universo disponível para a proposta de intervenção. Não foi realizado cálculo prévio do tamanho amostral, uma vez que a pesquisa teve caráter exploratório e ocorreu em um contexto específico, envolvendo o universo acessível de estudantes elegíveis. Contudo, a inclusão de todos os integrantes da turma assegurou a cobertura completa da população-alvo, atendendo ao objetivo de avaliar a viabilidade e os efeitos iniciais da estratégia educativa proposta.

### Randomização e cegamento

A alocação dos 32 estudantes entre o grupo-controle (GC) e o grupo experimental (GE) foi realizada por randomização estratificada (razão 1:1) para assegurar equilíbrio em características basais. Foram definidos estratos por sexo (masculino/feminino), faixa etária (intervalos de cinco anos) e turno de estudo (manhã/tarde). A sequência de randomização foi gerada por um estatístico independente, sem qualquer participação nas fases de recrutamento, intervenção ou coleta de dados. Para cada estrato, o estatístico utilizou a função *= ALEATÓRIO* ( ) do Microsoft Excel 2010 para gerar um valor numérico aleatório para cada participante; os participantes dentro de cada estrato foram ordenados por esse valor. Seguindo essa ordenação, as alocações foram atribuídas na proporção 1:1 (Grupo A = GE; Grupo B = GC).

A sequência final foi codificada (Grupo A/Grupo B) e armazenada em arquivo eletrônico protegido por senha, de acesso restrito exclusivamente ao estatístico gerador. A ocultação da alocação foi assegurada pelo procedimento centralizado: os pesquisadores que inscreveram os participantes solicitaram que o estatístico os alocasse somente após confirmação da elegibilidade e assinatura do TCLE; o estatístico devolvia apenas o código alocacional (A ou B) no momento da atribuição. Dessa forma, a equipe de recrutamento, os intervenientes e os responsáveis pela condução das atividades não tiveram acesso prévio à lista de alocação.

Quanto ao cegamento, os responsáveis pela análise dos dados e pela elaboração do material didático não participaram das intervenções nem da coleta de dados; assim, garantiu-se o que se chama “cegamento do avaliador” (*assessor-blind*). Os participantes não foram informados sobre o caráter comparativo entre os grupos (cegamento parcial dos participantes), e os profissionais que ministraram as intervenções atuaram sem acesso à composição dos grupos. Em resumo, o estudo adotou randomização estratificada com sequência gerada por estatístico independente e ocultação centralizada da alocação; o cegamento foi mantido para avaliadores/analisadores, com cegamento parcial dos participantes, mas não dos executores da intervenção.

Em conformidade com CONSORT, relatamos explicitamente: (1) método de geração da sequência, função *= ALEATÓRIO* ( ) do Excel, por estatístico independente; (2) mecanismo de ocultação, arquivo codificado e protegido e comunicação da alocação pelo estatístico somente após inclusão; (3) implementação - o estatístico gerou e guardou a lista, os pesquisadores inscreveram os participantes, a alocação foi comunicada pelo estatístico, e as intervenções foram aplicadas por equipe sem acesso à lista.

### Instrumentos utilizados para coleta de dados e variáveis

A coleta de dados utilizou um questionário sociodemográfico, um teste de conhecimento teórico^(15)^, um cenário simulado com checklist^(16)^ para avaliação de habilidades e a Escala de Avaliação do *Debriefing* Associado à Simulação (EADaS)^(17)^.

O questionário sociodemográfico incluiu informações sobre idade, sexo, raça, período do curso, formação prévia em saúde e contato com o tema. O teste de conhecimento teórico, com 20 questões sobre prevenção e manejo de LP, permitiu as respostas “Verdadeiro (V)”, “Falso (F)” ou “Não Sei (NS)”, com 1 ponto por acerto. Na validação, o teste apresentou valor de alfa de Cronbach de 0,85.

A simulação foi realizada com base em um cenário clínico estruturado e validado no tocante ao conteúdo sobre tratamento de LP em um ambiente de cuidados clínicos de um hospital. O cenário estruturado apresentou Índice de Validade de Conteúdo (IVC) global de 0,85. Já o checklist^(16)^, para avaliação das habilidades no cenário, contém 13 itens, que permitem verificar a capacidade do estudante de identificar, avaliar, selecionar e indicar a cobertura apropriada para lesão. Cada item é analisado pelo desempenho do estudante, com as opções: Não realizou (0 ponto), Realizou parcialmente (0,5 ponto) e Realizou adequadamente (1 ponto). Para atingir a média mínima de 70%, o estudante deveria obter 9,1 pontos em 13^(15)^. Cabe destacar que o checklist é um dos itens que compõe o cenário clínico estruturado.

Para avaliar o impacto do *debriefing* sob a perspectiva dos participantes, foi utilizado o instrumento EADaS^(17)^, composto por 32 itens distribuídos em três dimensões: Valor psicossocial, Valor cognitivo e Valor afetivo. A escala obteve um coeficiente alfa de Cronbach de 0,899, indicando alta consistência interna.

A principal variável de desfecho foi a adequada abordagem às LPs, mensurada por meio de um checklist durante a simulação. Como variáveis secundárias de desfecho, consideraram-se o conhecimento teórico e o impacto do *debriefing*, avaliados pelo teste teórico de conhecimento e pelo EADaS, respectivamente.

### Protocolo do estudo: intervenção e coleta de dados

O estudo foi desenvolvido em seis etapas: 1) Coleta dos dados sociodemográficos e avaliação do conhecimento antes da intervenção educativa (pré-teste); 2) Abordagem teórica sobre LP; 3) Treinamento de habilidades em um cenário com fotografias clínicas sobre LP para GC e GE; 4) Intervenção: aplicação do cenário clínico e avaliação das habilidades pelo checklist; 5) Realização do *Debriefing* e aplicação da EADaS; e 6) Avaliação do conhecimento (pós-teste).

Na primeira etapa, os estudantes responderam a um questionário sociodemográfico e realizaram a avaliação inicial do conhecimento teórico sobre LP (pré-teste), em um ambiente reservado, com duração de 30 minutos. Na segunda etapa, o conteúdo teórico foi ministrado ao longo de oito horas por profissionais estomaterapeutas com experiência em assistência e ensino. As aulas abordaram três grandes temas: Introdução a feridas, Introdução às LPs e Avaliação e tratamento das lesões. Essa etapa ocorreu no mesmo dia da primeira etapa. No dia seguinte à abordagem teórica, ocorreu a terceira etapa, momento em que os participantes passaram por um treinamento prático utilizando fotografias de LPs. O treinamento, idêntico para ambos os grupos, teve duração de duas horas e visou familiarizar os estudantes com a classificação e avaliação dessas lesões.

A quarta etapa, aplicada 14 dias após a primeira, foi o momento da intervenção, em que houve a simulação clínica e a avaliação das habilidades adquiridas. As duas semanas de intervalo foram adotadas para minimizar a possibilidade de os participantes lembrarem as respostas do teste anterior (viés de memória), garantindo que o desempenho no segundo teste reflita a retenção real do conhecimento. Nessa etapa, quatro enfermeiros (dois para o GE e dois para o GC) conduziram as orientações no cenário clínico e a avaliação, empregando um checklist validado. A etapa iniciou-se com um *briefing* de 5 minutos, seguido por 12 minutos para cada estudante desenvolver as atividades no cenário simulado.

A intervenção aplicada ao GE consistiu em um cenário de alta fidelidade, caracterizado por maior realismo e interatividade. Nesse intuito, utilizou-se a simulação clínica com um ator treinado para representar um paciente e interagir com os estudantes; além disso, foi feita a aplicação de feridas artificiais (*moulage*) sobre a pele e a apresentação de casos clínicos. Já o GC foi submetido a um cenário de média fidelidade que usou o manequim Simulador de Estágios de Lesões por Pressão TZJ-4009-U, também associado a casos clínicos. Dessa forma, o cenário de alta fidelidade, ao incluir um paciente simulado/ator com liberdade para a encenação, proporcionou uma experiência mais realista e imersiva em comparação com o ambiente de média fidelidade, que apresentou menor nível de interatividade.

Cabe destacar que tanto os enfermeiros quanto o ator passaram por um treinamento específico, cuidadosamente estruturado e padronizado, seguindo um protocolo detalhado. Esse treinamento foi desenvolvido para garantir que todos os envolvidos na intervenção tivessem uma compreensão clara dos objetivos, das ações a serem realizadas e dos procedimentos necessários. Os enfermeiros do GE receberam o treinamento separadamente dos enfermeiros do GC, para evitar interferências. Além disso, foi adotada a padronização do estilo de condução das atividades, contemplando linguagem utilizada, tempo destinado a cada etapa e forma de fornecer *feedback*. A finalidade foi minimizar eventuais diferenças individuais entre os instrutores e assegurar a equivalência da entrega pedagógica entre os grupos. Essa calibração minuciosa e coordenada visou assegurar que a intervenção ocorresse de maneira controlada, com o máximo de fidelidade ao protocolo estabelecido, proporcionando uma experiência de aprendizagem imersiva e de alta qualidade para os participantes.

A quinta etapa (*debriefing*) ocorreu ao final da intervenção educativa, sendo realizada separadamente com os estudantes do GE e do GC, em ambientes distintos, conforme a designação de cada grupo. Os participantes foram conduzidos à sala de *debriefing*, onde permaneceram até a formação completa do grupo. Com duração de 30 minutos, essa etapa foi conduzida pelos mesmos enfermeiros responsáveis pela avaliação das habilidades no cenário clínico, seguindo um roteiro estruturado com instruções norteadoras^(18)^. Ao término, os estudantes foram convidados a responder à EADaS^(17)^, com um tempo médio de 20 minutos.

A sexta e última etapa consistiu na aplicação do pós-teste para avaliar o conhecimento teórico sobre LPs. Os estudantes responderam ao mesmo teste aplicado na primeira etapa, em um ambiente reservado, com duração de 30 minutos.

As seis etapas totalizaram cerca de 12 horas de atividades por participante. Todos os elementos da intervenção foram previamente padronizados por meio de treinamentos específicos para os enfermeiros e para o ator, com o objetivo de garantir a fidelidade do protocolo e a consistência das avaliações.

### Análise dos dados

Os dados foram tabulados no *Microsoft Excel* ^®^ e analisados no software R 4.3.0. As variáveis categóricas foram descritas em frequências absolutas e relativas, ao passo que as variações numéricas foram descritas por meio de média, intervalo de confiança de 95% (IC 95%), mediana, desvio-padrão, quartis e valores máximo e mínimo. A consistência interna foi avaliada empregando-se Cronbach e ômega de McDonald, e as correlações entre itens foram feitas com o uso do coeficiente de Pearson.

Comparações entre GE e GC foram obtidas por meio do qui-quadrado (ou Fisher) para variáveis categóricas e mediante teste *t* ou Mann-Whitney para as numéricas, conforme a distribuição. Diferenças ao longo do tempo foram comprovadas com modelos Anova mistos.

Medidas de tamanho de efeito (V de Cramér, *d* de Cohen, *r*, e η^2^) foram calculadas para avaliar a magnitude dos resultados, com um nível de significância de 5%.

## RESULTADOS

Dos 36 estudantes elegíveis, 32 concluíram o protocolo de intervenção. A randomização permitiu alocar 17 estudantes no GE e 15 estudantes no GC, sem registro de perda no seguimento (Figura 1).

**Figura 1:** Diagrama do Ensaio Clínico Randomizado de acordo com *Consolidated Standards of Reporting Trials* (CONSORT)^(14)^ (n = 32), Minas Gerais, Brasil, 2023

Dentre os estudantes elegíveis, a maioria era do sexo feminino (71,9%) e apresentava idade média de 22,56 anos (DP = 1,80; IC 95%: 21,87-23,25). Os grupos eram homogêneos, não diferindo quanto às características sociodemográficas e ao contato prévio com a temática (Tabela 1).

**Tabela 1:** Comparação das variáveis sociodemográficas e do conhecimento prévio entre os grupos experimental e controle (N = 32), Minas Gerais, Brasil, 2023

Variável	Grupo		Valor de *p*
	Grupo-controle (n = 15)	Grupo experimental (n = 17)	
Idade (DP^*^)	22,67 (1,80) IC 95%^†^: 21,56-23,77	22,47 (1,91) IC 95%^†^: 21,25-23,68	0,768‡
**Sexo - n (%)**			
Feminino	11 (73,33)	12 (70,59)	1,000^§^
Masculino	4 (26,67)	5 (29,41)	
**Raça - n (%)**			
Branco	10 (66,67)	10 (58,82)	
Pardo	3 (20,00)	5 (29,41)	0,875^§^
Preto	2 (13,33)	2 (11,76)	
**Possui formação de nível técnico em saúde - n (%)**			
Não	14 (93,33)	17 (100,00)	0,469^§^
Sim	1 (6,67)	0 (0,00)	
**Contato prévio com a temática - n (%)**			
Não	15 (100,00)	17 (100,00)	0,456^§^
Sim	0 (0,00)	0 (0,00)	

* Desvio-padrão; †Intervalo de confiança de 95%;

‡ Teste t para amostras independentes; §Teste exato de Fisher.

Das 20 questões empregadas para avaliar o conhecimento teórico, a mediana foi 12 para o GC e 13 para o GE no pré-teste; já no pós-teste, ambos os grupos apresentaram mediana de 15 (Tabela 2).

**Tabela 2:** Estatística descritiva dos acertos de cada questão do questionário de conhecimento, de acordo com o grupo e momento no pré-teste e pós-teste (N = 32), Minas Gerais, Brasil, 2023

Variável	Pré-teste		Pós-teste	
	Grupo-controle (n = 15)	Grupo experimental (n = 17)	Grupo-controle (n = 15)	Grupo experimental (n = 17)
Quantidade de acertos				
Média (DP^*^)	12,47 (2,61)	12,12 (1,96)	15,36 (1,36)	15,07 (2,37)
IC 95%^†^	11,06-13,87	10,99-13,26	14,53-16,18	13,84-16,31
Mediana (Q1 - Q3)‡	12,00 (10,50 - 15,00)	13,00 (10,00 - 14,00)	15,00 (14,00 - 16,00)	15,00 (13,25 - 16,75)
Mín. - Máx.^§^	8 - 16	8 - 15	14 - 18	11 - 19

* Desvio-padrão; †Intervalo de confiança de 95%;

‡ Q1 = primeiro quartil (percentil 25); Q3 = terceiro quartil (percentil 75); §Valores mínimo e máximo observados.

A análise da ANOVA mista revelou um aumento significante na quantidade de acertos do pré-teste para o pós-teste, independentemente do grupo. Esse efeito é evidenciado pelo resultado estatístico para o fator Momento (F(1,23) = 25,298; p < 0,001; η^2^g = 0,333), indicando uma mudança expressiva e relevante na variável dependente ao longo do tempo.

Por outro lado, o fator Grupo não apresentou efeito significante sobre a quantidade de acertos (F (1,23) = 0,012; p = 0,914; η2g = 0,000), sugerindo que não houve diferença entre os grupos em termos de desempenho geral. Além disso, não foi identificada interação significante entre Grupo e Momento (F (1,23) = 0,154; p = 0,698; η2g = 0,003), indicando que o aumento na quantidade de acertos do pré-teste para o pós-teste ocorreu de forma semelhante em ambos os grupos, sem variações atribuíveis à interação entre essas variáveis.

Conclui-se, portanto, que a melhoria observada na quantidade de acertos deve-se ao fator temporal (momento da avaliação), sem influência significante do grupo ou da interação entre grupo e momento. Esses achados indicam que ambos os grupos apresentaram um desempenho semelhante ao longo do tempo, sugerindo que outros fatores, além da diferenciação entre os grupos, podem ter contribuído para o aumento dos acertos. Ademais, a consistência interna do teste de conhecimento sobre LP foi adequada, com um alfa de Cronbach de 0,705 e um ômega de McDonald de 0,798, reforçando a confiabilidade da avaliação utilizada.

Quanto à avaliação das habilidades no cenário clínico simulado, as notas gerais foram muito próximas entre os grupos, com médias de 10,91 (GC) e 10,79 (GE). O GE demonstrou desempenho superior em itens como o 2 (escolha das coberturas) e o 11 (bordas da ferida), mas apresentou dificuldades maiores em outros, como o Item 5 (localização anatômica). O GC, por outro lado, teve desempenho mais homogêneo, exceto pelo Item 9 (classificação da dor) (Tabela 3).

**Tabela 3:** Análise descritiva das variáveis referentes à habilidade no cenário clínico simulado, de acordo com o grupo (N = 32), Minas Gerais, Brasil, 2023

Variável	Grupo (N = 32)	
	Grupo-controle (n = 15) - n (%)	Grupo experimental (n = 17) - n (%)
Item 1 - Os estudantes identificaram e classificaram o estágio da lesão por pressão?		
Acerto	12 (80,00)	15 (88,30)
Erro	3 (20,00)	2 (11,70)
**Item 2 - Os estudantes selecionaram e relataram as coberturas adequadas?**		
Acerto	13 (86,70)	17 (100,00)
Erro	2 (13,30)	0 (0,00)
**Item 3 - Os estudantes justificaram a escolha (indicação) das coberturas?**		
Acerto	13 (86,70)	14 (83,35)
Erro	2 (13,30)	3 (17,65)
**Item 4 - Os estudantes orientaram o paciente/familiar sobre o curativo e outros procedimentos que implicam a melhora do quadro geral da LP?**		
Acerto	15 (100,00)	17 (100,00)
Erro	0 (0,00)	0 (0,00)
**Item 5 - Os estudantes identificaram e classificaram a localização anatômica da lesão por pressão?**		
Erro	3 (20,00)	12 (70,60)
Acerto	12 (80,00)	5 (29,40)
**Item 6 - Os estudantes mensuraram o tamanho (comprimento, largura e profundidade) da lesão?**		
Acerto	15 (100,00)	17 (100,00)
Erro	0 (0,00)	0 (0,00)
**Item 7 - Os estudantes avaliaram e classificaram os tecidos presentes no leito da lesão por pressão?**		
Acerto	15 (100,00)	16 (94,10)
Erro	0 (0,00)	1 (5,90)
**Item 8 - Os estudantes identificaram e classificaram os tipos de tecido?**		
Acerto	15 (100,00)	16 (94,10)
Erro	0 (0,00)	1 (5,90)
**Item 9 - Os estudantes identificaram e classificaram a dor?**		
Erro	13 (86,70)	12 (70,60)
Acerto	2 (13,30)	5 (29,40)
**Item 10 - Os estudantes identificaram e classificaram a condição da pele ao redor da lesão?**		
Acerto	12 (80,00)	14 (83,35)
Erro	3 (20,00)	3 (17,65)
**Item 11 - Os estudantes identificaram e classificaram as bordas da ferida?**		
Acerto	10 (66,70)	15 (88,30)
Erro	5 (33,30)	2 (11,70)
**Item 12 - Os estudantes identificaram e classificaram os aspectos do exsudato?**		
Acerto	13 (86,70)	12 (70,60)
Erro	2 (13,30)	5 (29,40)
**Item 13 - Os estudantes realizaram a anotação de enfermagem?**		
Acerto	11 (100,00)	12 (70,60)
Erro	0 (0,00)	5 (29,40)

A análise inferencial revelou que não houve diferença estatisticamente significante na quantidade de acertos no teste de habilidades entre o GE e o GC (p = 0,853; tamanho do efeito pequeno, d = 0,076). As médias das notas foram semelhantes entre os grupos, com 10,91 (DP ± 1,64; IC 95%: 9,96-11,86) no GC e 10,79 (DP ± 1,63; IC 95%: 9,88-11,71) no GE. Da mesma forma, as medianas foram equivalentes, registrando 11,00 (Q1 = 10,00; Q3 = 12,00) no GC e 10,50 (Q1 = 10,00; Q3 = 12,00) no GE.

Esses resultados sugerem que, apesar das intervenções, não houve diferenças significantes entre os grupos na execução das habilidades avaliadas. Independentemente do cenário ser de alta ou média fidelidade, os desempenhos foram elevados e consistentes, indicando que ambos os grupos alcançaram níveis satisfatórios na realização das atividades no ambiente clínico simulado.

Quanto à avaliação global da EADaS, o GE apresentou uma média ligeiramente superior (121,08±11,22; IC95%: 110,46-121,19) em comparação ao GC (115,82±9,03; IC95%:114,99-127,18). Contudo, a diferença não foi significante (p = 0,2251), e o tamanho de efeito foi pequeno (d = -0,511). Esses resultados revelam que ambos os grupos tiveram experiências semelhantes em relação ao *debriefing*, sem diferenças claras nas percepções psicossociais, cognitivas, afetivas ou no geral (Tabela 4).

**Tabela 4:** Comparação dos escores de cada fator e do escore geral Escala de Avaliação do *Debriefing* Associado à Simulação entre o grupo-controle e o experimental (N = 32), Minas Gerais, Brasil, 2023

Dimensões	Grupo		Valor de *p*	TE^†^
	Grupo-controle (n = 15)	Grupo experimental (n = 17)		
Valor psicossocial			0,080^*^	-0,737
Média (DP^‡^)	54,27 (7,20)	58,64 (4,73)		
IC 95%^§^	49,97-58,57	56,14-61,14		
Mediana (Q1; Q3^||^)	53,00 (51,00; 58,50)	60,00 (57,50; 62,00)		
**Valor cognitivo**			0,978^#^	-0,011
Média (DP^‡^)	42,27 (3,29)	42,57 (2,41)		
IC 95%^§^	40,45-44,09	41,25-43,89		
Mediana (Q1; Q3^||^)	44,00 (40,00; 44,50)	43,50 (41,00; 44,75)		
**Valor afetivo**			0,839^#^	0,048
Média (DP^‡^)	19,27 (6,34)	19,77 (8,22)		
IC 95%^§^	15,79-22,75	15,45-24,09		
Mediana (Q1; Q3^||^)	18,00 (14,00; 24,00)	19,00 (15,00; 20,00)		
**Geral nas três dimensões**			0,225^*^	-0,511
Média (DP^‡^)	115,82 (9,03)	121,08 (11,22)		
IC 95%^§^	110,46-121,19	114,99-127,18		
Mediana (Q1; Q3^||^)	115,00 (110,50; 121,00)	119,00 (114,00; 125,00)		

* Teste t para amostras independentes;

† TE - Tamanho de efeito; Foram calculados os seguintes tamanhos de efeito: d de Cohen, para o teste t em amostras independentes; r, para o teste de Mann-Whitney; ‡Desvio-padrão; §Intervalo de confiança de 95%;

|| Q1 = primeiro quartil (percentil 25); Q3 = terceiro quartil (percentil 75);

# Teste de Mann-Whitney.

## DISCUSSÃO

No presente estudo, a maioria dos participantes eram mulheres jovens, brancas, com algum conhecimento prévio sobre a temática. Esses dados são compatíveis com os resultados de outras pesquisas que utilizaram a simulação para ensinar o manejo da LP a estudantes de graduação em Enfermagem^(19-21)^. Em outro estudo^(22)^ experimental, que também empregou a simulação clínica para esse fim, a idade média era de 21,44 (±2,37) anos. Entre eles, 45,7% eram do sexo feminino, e 76,5% tinham algum conhecimento prévio sobre a temática.

Os resultados indicaram uma melhora significante no conhecimento teórico dos estudantes entre o pré-teste e o pós-teste em ambos os grupos, sugerindo que essa evolução está associada à atividade educativa teórica, aliada à simulação clínica. Contudo, uma revisão sistemática não identificou melhorias significantes no conhecimento dos estudantes com o uso de simulações clínicas^(21)^. Por outro lado, um estudo sobre simulações de alta e baixa fidelidade encontrou uma diferença estatisticamente significante entre o pré-teste e o pós-teste teórico em ambos os grupos, reforçando os achados desta pesquisa^(22)^.

Cabe destacar que o conhecimento teórico desempenha um papel essencial na eficácia da simulação clínica, pois fornece a base necessária para compreender conceitos que fundamentam o desempenho nas habilidades psicomotoras^(20,21,23)^. Esse conhecimento não apenas favorece a tomada de decisões rápidas e precisas, mas também potencializa a capacidade de resolver problemas de maneira mais eficiente, contribuindo para um desempenho clínico mais seguro e eficaz^(21)^. Além disso, o domínio teórico é crucial para que os estudantes possam interpretar cenários simulados de forma crítica, aplicar protocolos com assertividade e transferir o aprendizado para situações reais, fortalecendo sua confiança e competência profissional^(13,21-23)^. A integração entre teoria e prática é, portanto, indispensável para maximizar os benefícios das estratégias educacionais baseadas em simulação.

Em relação às habilidades clínicas para o manejo da LP, não foram identificadas diferenças estatisticamente significantes no desempenho do GE em relação ao GC. Apesar do maior realismo dos cenários de alta fidelidade (GE), os resultados indicam que o nível de fidelidade não influenciou o desempenho. Uma possível explicação para esse achado é a ocorrência de um “efeito teto”, dado o desempenho elevado e relativamente homogêneo entre os grupos, o que pode ter limitado a detecção de diferenças. Assim, refuta-se a hipótese de que a simulação de alta fidelidade seria mais eficaz que a de média fidelidade no estímulo às habilidades clínicas para o manejo de LP.

Um estudo^(24)^ comparou a eficácia de cenários de alta e média fidelidade no estadiamento de LP por estudantes de Enfermagem turcos. Ambos os grupos melhoraram no conhecimento teórico, mas o GE, que usou simuladores de alta fidelidade com *moulage* de feridas, obteve escores práticos e acertos significantemente superiores (p = 0,02). Nessa direção, uma revisão sistemática^(25)^ também destacou que a simulação de alta fidelidade favorece habilidades como pensamento crítico, julgamento clínico e tomada de decisão em estudantes de Enfermagem.

Por outro lado, estudos indicam que a eficácia do aprendizado na simulação depende mais do planejamento e da adaptação às necessidades dos estudantes do que do nível de fidelidade do cenário^(21,26)^. Fatores como objetivos claros, alinhamento com as competências, *briefing* e *debriefing* de qualidade e adequação dos cenários são mais determinantes para o sucesso do que o realismo tecnológico^(26-30)^.

No *debriefing*, etapa final da simulação clínica, os estudantes refletiram sobre suas ações e decisões, promovendo autoavaliação e integração entre teoria e prática. Utilizou-se um modelo estruturado, que permitiu revisão reflexiva, avaliação do desempenho e identificação de melhorias^(18)^. Uma revisão sistemática^(29)^ destacou que o *debriefing* aprimora raciocínio clínico, pensamento crítico, autoconfiança e autoeficácia em estudantes de Enfermagem.

Para avaliar a experiência do estudante com o *debriefing*, aplicou-se a EADaS^(17)^, que analisou as dimensões psicossocial, cognitiva e afetiva. Escores positivos foram observados em todas as dimensões, com destaque para autoconfiança, liderança, trabalho em equipe, reflexão cognitiva e gestão emocional. Contudo, não houve diferenças estatisticamente significantes entre o GE e o GC, indicando benefícios similares entre as intervenções.

### Limitações do estudo

Este estudo apresenta algumas limitações que devem ser consideradas na interpretação dos resultados. O reduzido número amostral e a condução em um único centro de treinamento com professores e instrutores específicos limitam a generalização dos achados, exigindo cautela na extrapolação para outras populações e contextos. Ainda assim, os dados obtidos fornecem indícios relevantes sobre o potencial da intervenção, podendo servir como base preliminar para a aplicação prática em cenários semelhantes.

Além disso, o curto período de acompanhamento, com o pós-teste realizado logo após a intervenção e sem reavaliação tardia das habilidades no cenário simulado, impediu a análise da durabilidade dos efeitos da intervenção. Tal limitação restringe a compreensão sobre a consolidação e manutenção do conhecimento e das habilidades ao longo do tempo.

Assim, embora os resultados sugiram benefícios da estratégia utilizada, é essencial que futuras investigações ampliem o tamanho amostral, incluam múltiplos centros de treinamento e variabilidade de professores/instrutores e adotem desenho longitudinal com seguimento de longo prazo (p.ex., 30 ou 90 dias), a fim de avaliar com maior robustez a eficácia e a sustentabilidade das intervenções educativas no contexto estudado.

### Contribuições para a Área

Os achados deste estudo reforçam a relevância de práticas educacionais baseadas em evidências, especialmente no campo da simulação clínica aplicada à formação em enfermagem. Ao comparar diferentes níveis de fidelidade na simulação, os resultados indicam caminhos promissores para o aperfeiçoamento das estratégias pedagógicas, destacando a viabilidade de intervenções com menor complexidade tecnológica, porém ainda eficazes na promoção do aprendizado. Essa perspectiva amplia o acesso a metodologias ativas em contextos com restrições de recursos, contribuindo para a democratização do ensino e sustentabilidade das práticas formativas. Mesmo diante das limitações, os dados oferecem subsídios importantes para a tomada de decisão no planejamento educacional, apoiando gestores e educadores na adoção de abordagens mais realistas e adaptáveis às diversas realidades da educação em enfermagem.

Para investigações futuras, sugere-se a incorporação de análises qualitativas dos *debriefings*, a fim de aprofundar a compreensão dos processos reflexivos dos estudantes e identificar elementos que possam potencializar o desenvolvimento de habilidades clínicas e críticas.

## CONCLUSÕES

O estudo permitiu comparar a eficácia da simulação clínica de alta e de média fidelidade para estudantes de Enfermagem na avaliação e tratamento de lesão por pressão. Quanto ao conhecimento teórico, foi verificada evolução significante entre o pré-teste e o pós-teste para ambos os grupos. Não foi verificada diferença estatística significante entre os grupos quanto ao desempenho das habilidades, com escores similares para os estudantes que desenvolveram as atividades no cenário de alta e média fidelidade. Além disso, a experiência com o *debriefing* foi considerada positiva e equivalente nos dois grupos, indicando que a reflexão crítica e o *feedback* imediato foram elementos centrais no aprendizado.

## Data Availability

Os dados de pesquisa estão disponíveis no corpo do artigo.
